# Pulmonary Toxicity of Instilled Silver Nanoparticles: Influence of Size, Coating and Rat Strain

**DOI:** 10.1371/journal.pone.0119726

**Published:** 2015-03-06

**Authors:** Joanna Seiffert, Farhana Hussain, Coen Wiegman, Feng Li, Leo Bey, Warren Baker, Alexandra Porter, Mary P. Ryan, Yan Chang, Andrew Gow, Junfeng Zhang, Jie Zhu, Terry D. Tetley, Kian Fan Chung

**Affiliations:** 1 Airways Disease, National Heart & Lung Institute, Imperial College, London, United Kingdom; 2 Department of Material Science, Chemistry and the London Centre for Nanotechnology, Imperial College, London, United Kingdom; 3 Department of Mechanical Engineering, Faculty of Engineering Building, University of Malaya, Kuala Lumpur, Malaysia; 4 Department of Preventive Medicine, Keck School of Medicine, University of Southern California, Los Angeles, California, United States of America; 5 Department of Pharmacology and Toxicology, Rutgers University, Piscataway, New Jersey, United States of America; 6 Nicholas School of Environment & Duke Global Health Institute, Duke University, Durham, United States of America; University ofTennessee Health Science Center, UNITED STATES

## Abstract

Particle size and surface chemistry are potential determinants of silver nanoparticle (AgNP) respiratory toxicity that may also depend on the lung inflammatory state. We compared the effects of intratracheally-administered AgNPs (20nm and 110nm; polyvinylpyrrolidone (PVP) and citrate-capped; 0.1 mg/Kg) in Brown-Norway (BN) and Sprague-Dawley (SD) rats. In BN rats, there was both a neutrophilic and eosinophilic response, while in SD rats, there was a neutrophilic response at day 1, greatest for the 20nm citrate-capped AgNPs. Eosinophilic cationic protein was increased in bronchoalveolar lavage (BAL) in BN and SD rats on day 1. BAL protein and malondialdehyde levels were increased in BN rats at 1 and 7 days, and BAL KC, CCL11 and IL-13 levels at day 1, with increased expression of CCL11 in lung tissue. Pulmonary resistance increased and compliance decreased at day 1, with persistence at day 7. The 20nm, but not the 110 nm, AgNPs increased bronchial hyperresponsiveness on day 1, which continued at day 7 for the citrate-capped AgNPs only. The 20nm versus the 110 nm size were more proinflammatory in terms of neutrophil influx, but there was little difference between the citrate-capped versus the PVP-capped AgNPs. AgNPs can induce pulmonary eosinophilic and neutrophilic inflammation with bronchial hyperresponsiveness, features characteristic of asthma.

## Introduction

Among the consumer products that contain engineered nanoparticles, one of the most common materials used is nanosilver [1, 2]. Silver nanoparticles (AgNPs) possess antimicrobial properties and have been utilised in commercial applications, ranging from medical products, such as wound dressings and disinfectant sprays to domestic applications, such as household cleaning agents, odour-resistant clothing, and personal hygiene products [3, 4]. More recently nanoparticle formulations have become favoured commercially due to their slow and stable release of silver ions and bacterial killing properties [5]. There is growing concern that the widespread use of AgNPs in consumer products may impact negatively on human health, especially when these nanoparticles can be inhaled by workers handling these nanoparticles, and by domestic users and patients exposed to medical products containing AgNPs.

The size and surface area of AgNPs are major determinants of pulmonary toxicity of AgNPs inhaled into the lungs that will affect bio-availability, surface charge and dissolution rates [6–11]. Synthesis of AgNPs generally involves the use of surface coatings or capping agents to prevent oxidation and dissolution of silver ions and agglomeration of particles. Two commonly-used coating agents are sodium citrate and polyvinylpyrrolidone (PVP), which impart a negative charge, giving them a wide appeal for manufacturing and consumer use.

Studies of the toxic effects of AgNPs in animals have mainly assessed the effects of oral or systemic exposure [12–14], with a limited number reporting the effects of inhalation and instillation into the lung. Clearance of particles administered by instillation is usually slower than that for inhaled particles [15], with a greater biological effect of instilled particles than inhaled particles [16]. Inhalation exposure of AgNPs in rodents induced mild dose-dependent pulmonary inflammation with transient changes in pulmonary function [17–19], while a single instillation of AgNPs into the lungs of mice caused a greater infiltration of leukocytes and elevated cytokine levels [20, 21]. *In vitro* studies on pulmonary cells indicate that AgNPs can damage mitochondria and increase reactive-oxygen species [ROS] [22], leading to mitochondrial-dependent apoptosis [23]. How these contribute to the development of pulmonary inflammation and alterations in lung function remain unclear.

In the current study, we chose to study a submaximal dose of AgNPs that has been shown to cause inflammation in rats [24] and that we ourselves have confirmed in preliminary studies in the BN rat to be inflammatory. We focused our study on the examination of the effect of two sizes of AgNPs of 20 and 110 nm in diameter with 2 different coatings either with citrate or PVP, and the time course effects of these AgNPs at 1, 7 and 21 days in the rat. We elected to study the Brown-Norway (BN) rat because this species is known to develop features of allergic asthma, namely lung eosinophilia and bronchial hyperresponsiveness (BHR) following sensitisation and exposure to allergens [25, 26]. To determine whether the pulmonary effects of intratracheally-instilled AgNPs administered at a single submaximal dose may be influenced by the pre-existing state of the lungs, we compared the responses of BN rats to the 20 nm AgNPs with those observed in the Sprague-Dawley (SD) rat [27].

## Materials and Methods

### Characterisation of AgNPs

20nm citrate-stabilised (Ag20Citrate), 20 nm PVP-stabilised (Ag20PVP), 110 nm citrate-stabilised (Ag110Citrate) and 110 nm PVP-stabilised (Ag110PVP) colloidal AgNPs were purchased from NanoComposix Inc. [San Diego, CA, USA]. The AgNPs were fabricated via base-catalysed reduction of silver nitrate onto a monodisperse 7nm gold seed. The citrate-coated AgNPs were provided in a 2mM citrate buffer, while the PVP-coated materials were suspended in water, and all were stored from light at 2–8°C. The NPs were characterised by Nanotechnology Characterisation Laboratory (NCL), National Cancer Institute, Frederick MD, USA (Table 1). We also characterised the solubility, stability, aggregate size and surface charge in water (pH 7, 37°C) and in the presence of the main constituent of lung surfactant, dipalmitoylphosphatidylcholine (DPPC), to reflect lung lining fluid. DPPC (100 μg/mL) was prepared by sonication for 10 minutes, adjusting to pH of 7 using either NaClO4 or HClO4. Each AgNP suspension (25 μg/mL; with and without DPPC) was incubated at 37°C for 1 day, then washed three times to remove excess organic surfactant and examined by inductively-coupled plasma-optical emission spectroscopy (ICP-OES), high annular dark field scanning transmission electron microscopy (HAADF-STEM), and energy dispersive X-ray analysis (EDS), as previously described (11).

**Table 1:** Characterisation of silver nanoparticles.

Silver nanoparticle [nm, capping agent]	Endotoxin [EU/mL]^a^	Hydro-dynamic diameter [nm]^b^	Diameter nanosphere[nm]^c^	Silver [mg/g]^d^	Free Ag^+^ [μg ion/g]^e^	Zetapotential [mV]^f^
Ag20PVP	<2.2	26.0	20.5	1.1	0.263± 0.048	−37
Ag20citrate	<0.03	24.0	20.3	1.1	0.100± 0.023	−48
110Ag PVP	<0.5	112.3	111.3	1.1	0.131 ± 0.021	−26
Ag110citrate	<0.03	104.2	111.5	1.0	0.071± 0.021	−43

^a^ Measured by kinetic turbidity and gel-clot Limulus Amoebocyte Lysate assay

^b^ Measured by dynamic light scattering

^c^ Measured by transmission electron microscopy [JOEL 2010 TEM]

^d,e^ Measured by inductively coupled plasma-Optical emission [ICP-OES]

^f^ Measured by ZetaPALS [Brookhaven Instruments Corporation, Holtsville, NY].

### Intratracheal instillation of AgNPs

The experiments were performed within the legal framework of the United Kingdom under a Project License granted by the Home Office of Her Majesty's government. The researchers hold Personal Licenses provided by the Home Office to perform the experiments in the rat species described here (Project Licence number: PPL 70/7581).

Instillation of AgNP suspensions or control solutions into the trachea of BN rats (male; 300–400g; 8–12 weeks old) and SD rats (250–350 g; 8–12 weeks) was performed under isoflurane anaesthesia (3.5 minutes/3.5% isofluorane /3.5 O_2_). BN rats were instilled with 0.3 mL of 1:10 dilutions of the 1.0 mg/mL of each of the 4 AgNP suspensions, equivalent to 0.1 mg/kg body weight. SD rats were administered only the 20nm AgNP suspensions. BN rats were instilled with control solutions of 0.3 mL of either distilled water or 1mM citrate (pH7.4), or suspensions of polyvinylpyrrolidone (PVP) in water. Regarding the PVP Polymers, the 10 kDa PVP (ISP Technologies, Inc.) was used as a control for the PVP-coated 20 nm AgNPs at a concentration of 33 μg/ml, and the 40 kDa, PVP (Calbiochem) was used as a control for the PVP-coated 110 nm AgNPs at a concentration of 62 μg/ml.

### Lung mechanics and BHR

1, 7 or 21 days after instillation of AgNPs, lung mechanics was assessed in anaesthetised (hypnorm/hypnovel i.p) and tracheotomised rats by the forced oscillation technique, using a computer-controlled animal ventilator (eSpira; EMMS, UK) at a tidal volume of 10 mL/Kg, a frequency of 90 breaths/minute and with a positive end-expiratory pressure (PEEP) of 5 cm H_2_O. Lungs were inflated three successive times to total lung capacity (TLC) at 30 cm of H_2_O. Resistance (Rrs) and dynamic compliance (Crs) of the whole respiratory system were assessed using a 1.2 second single frequency sinusoidal oscillation, termed a ‘snapshot’ manoeuvre. The mean value of 13 snapshot manoeuvres during a 3-minute period, was calculated. Rrs was recorded at baseline and following a 5-second exposure to increasing doubling concentrations of an acetylcholine (ACh) aerosol (0, 4, 8, 16, 32 and 64 mg/ml) from a nebulizer (Aeroneb, EMMS, Hants, UK). From the plot of Rrs versus log ACh concentration, the area under the curve (AUC) was calculated [28], and used as a measure of airway responsiveness.

### Bronchoalveolar lavage

After sacrifice by sodium pentobarbital, bronchoalveolar lavage (BAL) was performed via the tracheostomy tube with two 5.5 mL aliquots of ice-cold PBS. BAL cells were pelleted by centrifugation, the supernatants were snap-frozen at −80°C, and cytospin slides were prepared with 100,000 cells on each slide. Staining for differential cell count was performed using the DiffQuick kit (Polysciences Inc). At least 300 cells were counted. BAL supernatants were assayed for total protein (Biorad, Hemel Hempstead, UK), KC and ECP by ELISA kits (R & D, Abingdon, UK and Cusabio Biotech, Newmarket, Suffolk, UK, respectively), and for IL-13 and IgE by ELISA (Abcam, Cambridge, UK). IL-5 and CCL11 were also assayed by ELISA kits (Cusabio Biotech). BAL malondialdehyde (MDA) was measured using a HPLC system with fluorescent detection (Waters, Milford, MA, USA). A Nova-Pak C18 column (Waters) was used with a mobile phase of 40% methanol and 60% water.

### Lung histological analysis

The left lung was inflated with fresh 4% paraformaldehyde and was then processed using a histological automatic tissue processor and embedded in paraffin. Paraffin blocks were sectioned to expose the maximum surface area of lung tissue in the plane of the bronchial tree. Sections (5 μm) were then cut and stained with hematoxylin and eosin (BDH, Lutterworth, U.K.). For the detection of eosinophils, lung sections were stained using Carbol Chromotrope stain, which was made from Chromotrope 2R (BDH, Poole, UK). Sections were counterstained with haematoxylin and mounted with DPX mounting medium under glass coverslips.

The severity of inflammatory response observed in the hematoxylin and eosin-stained lung sections was scored on a 0–3 scale defined as: 0 no inflammatory response; 1 mild inflammation with foci of inflammatory cells in bronchial or vascular wall and in alveolar septa; 2 moderate inflammation with patchy inflammation or localized inflammation in walls of bronchi or blood vessels and alveolar septa, and less than one-third of lung cross-sectional area is involved; and 3 severe inflammation with diffuse inflammatory cells in walls of bronchi or blood vessels and alveoli septa; between one-third and two-thirds of the lung area is involved.

### Extraction of Protein and RNA from lung tissue and PCR

Total protein from cytosolic fraction was extracted from the upper lobes. Briefly, lung tissue was homogenised, followed by the addition of 0.5% NP40 and centrifugation at 7000 rpm for 5 minutes at 4°C. ECP in the tissues was measured by ELISA.

Total RNA was isolated using the RNeasy kit (Qiagen). RNA was reverse-transcribed into cDNA using a cDNA Reverse Transcription kit (Applied Biosystems, CA 94404, USA) and a RoboCycler (Stratagene, USA). cDNA was amplified by quantitative real-time polymerase chain reaction (PCR) using the SYBR PCR Kit (Qiagen) in a Rotor Gene 3000 (Corbett Research, Australia). Forward and reverse primers for PCR were: for rat Interferon-γ: F5’-AGCATGGATATGGAAGG-3’ and R5’-CGTATGGCCTGGTTGTCTTT-3’; rat CCL11 (eotaxin): F5’-TGCTGCTTTACCATGACCAG-3’ and R5’-CTTTTTCTTGGGCTTGGGGTCAGCAC-3’. Primers for IL-5 were obtained from the pre-optimised rat Rn IL-5_1_SG QuantiTect primer assay kit (Qiagen; gene ID 24497) and 18S/28S forward and reverse primers (gene ID 24721). Primers were used at a concentration of 0.5μM and cycling conditions were: step 1, 15 min at 95°C to activate the DNA Polymerase followed by cycling for 15 sec at 94°C, 30 S at 60°C and 30 sec at 72°C, repeated for 50 cycles. The relative expression of the genes were calculated from standard curves and normalised to 18s RNA.

### Data analysis

Data are reported as mean ± SD. All data were assessed for normality using the Shapiro-Wilk normality test. As the data was not normally distributed, comparison of the means of the multiple groups at the 3 different time-points was performed by the Kruskal-Wallis test using Prism 5 statistical package. Any differences between the individual groups were assessed by Dunn’s post-hoc test. A p value of 5% or less was taken as significant.

## Results

### Characterisation of AgNPs

**Fig 1:**
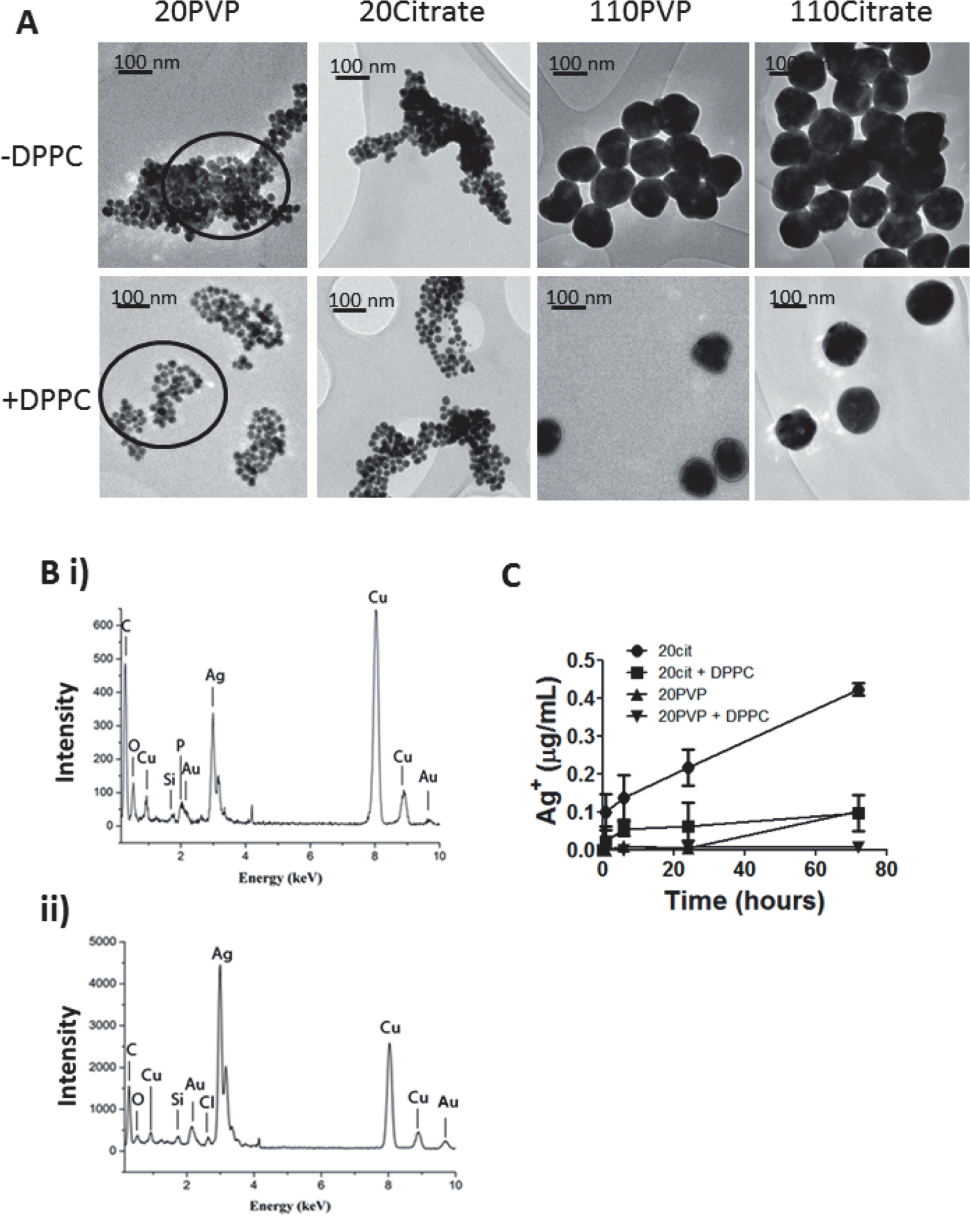
Characterisation of silver nanoparticle size, stability, composition and solubility. Panel A: TEM images of 20 nm and 110 nm silver nanoparticles either PVP- or citrate-capped following a 24 hour incubation in water (pH7, 37°C) in the absence (-) and presence (+) of dipalmitoylphosphatidylcholine (DPPC). Panel B: EDS spectra of (i) Ag20PVP without DPPC (First circle in upper part of Panel A) and (ii) Ag20PVP with DPPC (First circle in lower part of Panel A). Panel C: Time-dependent dissolution of 20 nm silver nanoparticle citrate- or PVP-capped suspensions (25 μg/mL) in water (pH7, 37°C), or in water (pH7, 37°C) plus DPPC. Ion concentrations are shown as μg/mL as determined by ICP-MS. Data shown as mean ± SD of three incubations.

TEM images confirmed that the AgNPs tended to agglomerate following incubation in water for 24 hours at 37°C, pH 7; denser agglomerates were observed for the 20nm compared with the 110nm particles (**Fig. 1A**). Addition of dipalmitoylphosphatidylcholine (DPPC) reduced agglomeration for all AgNPs, with greatest effects on the 110nm particles. EDS spectra, taken under identical acquisition conditions, confirmed a higher local concentration of silver from within the aggregates before and after the addition of DPPC (**Fig. 1B**).

Both 110nm particles (25 μg/mL, pH 7) showed negligible dissolution in H_2_O over 200 hours (data not shown), while dissolution of the 20 nm particles was dependent on the type of coating agent and the presence of DPPC (**Fig. 1C**). Ag20PVP NPs showed less dissolution than the Ag20citrate. Addition of DPPC lowered the dissolution rate of the 20nm NPs to 0.05% (PVP) and 1.1% (citrate) of the total mass of silver.

### Effect of dissolved silver ions

In order to determine the potential effect of solubilised silver, we instilled the maximum amount of silver ions that would be solubilised under the *in vitro* conditions, as shown in Table 1. Thus, SD rats were instilled with 1μg of silver ions in the form of silver nitrate. We found no significant effect in terms of total cell, neutrophil, eosinophil, macrophage and lymphocyte counts in BAL at 1, 7 or 21 days. Total protein and MDA levels in BAL also did not change compared to control. There were no changes in lung resistance or dynamic compliance, and no changes in bronchial responsiveness (*data not shown*).

### BAL and lung cellular responses

**Fig 2:**
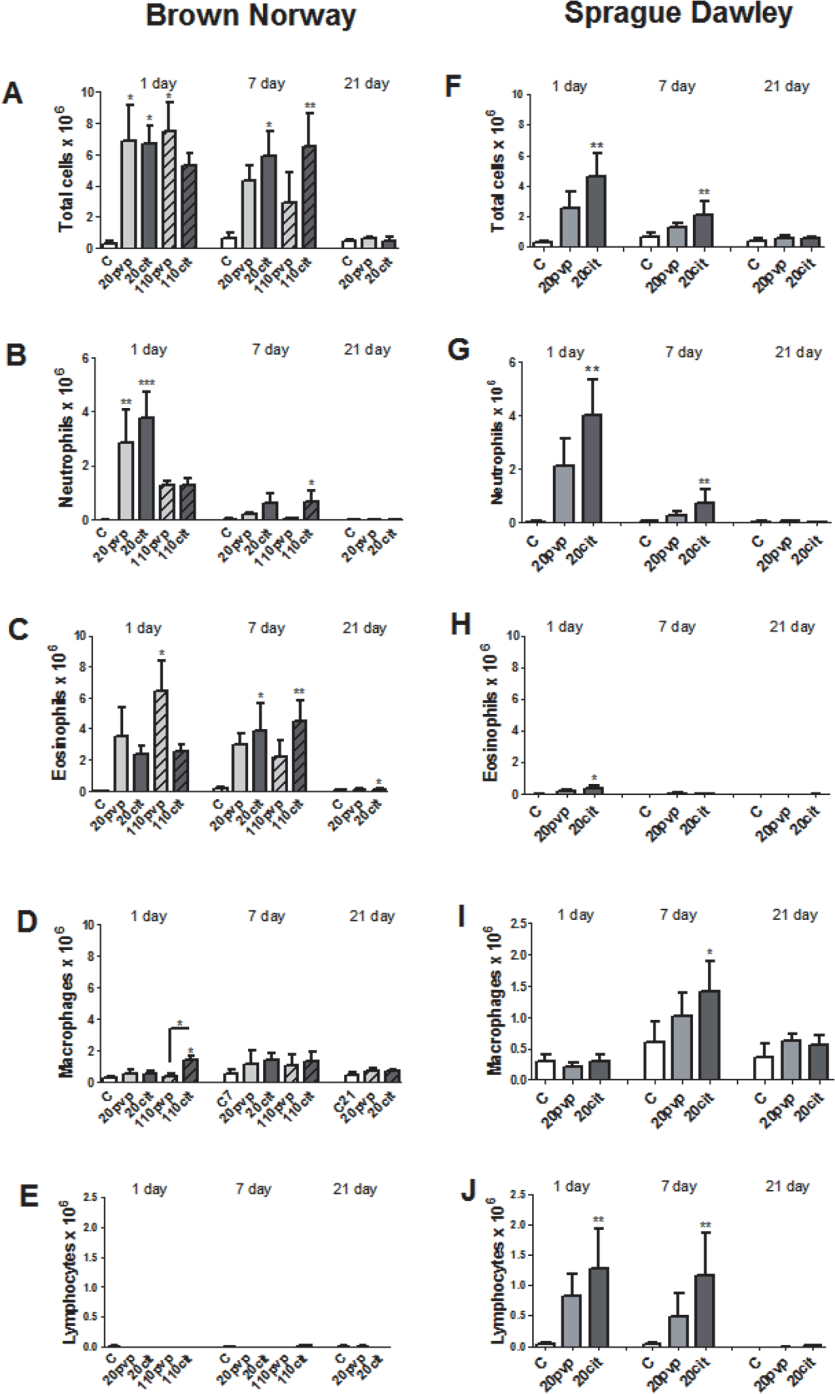
Bronchoalveolar lavage cells from rats at 1, 7 and 21 days after intratracheal instillation of silver nanoparticles (0.1 mg/kg). The effects of 20nm and 110 nm silver nanospheres capped with citrate (20cit, 110cit) or polyvinlypyrrolidone (20pvp, 110pvp) in Brown Norway rats (Panels A, B, C, D & E) and of 20 nm silver nanoparticles capped with citrate or pvp in Sprague Dawley rats (Panels F, G, H, I & J) are shown. Data expressed as mean ± SD, n = 5–6 for each group. *P<0.05, **P<0.001, ***P<0.0001 versus the water control (C) within each time-point; +P<0.05.

Exposure of BN rats to distilled water, 1mM citrate, 10 kDa PVP or 40 kDa PVP did not alter BAL cell numbers. In the BN rat, all AgNPs increased total cells (**Fig. 2A**) at 1 day apart from Ag110cit. Neutrophil influx was only increased by the 20 nm AgNPs (**Fig. 2B**). The type of coating was also important for persistent neutrophilic inflammation at 7 days and while the number of neutrophils fell to non-significant levels for the Ag20PVP and Ag110PVP particles, neutrophils induced by the citrate-coated 110 nm Ag particles remained elevated. At 1 day, there was only a significant increase in eosinophils for Ag110pvp that disappeared by day 7 (**Fig. 2C**), with increases for the 20 and 110 nm citrate-coated NPs appearing at 7 days Following Ag110citrate instillation, there was also a significant increase in macrophages at day 1 (**Fig. 2D**). There was no effect on lymphocytes (**Fig. 2E**). In SD rats, only Ag20citrate induced significant increase in total cells (**Fig. 2F**) and neutrophilia (**Fig. 2G**) at day 1 that persisted at day 7.Eosinophilic recruitment was also most evident with Ag20citrate (**Fig. 2H**) although BAL numbers were much lower compared to BN rats (**Fig. 2G,H**). This eosinophilic response subsided by day 7, and disappeared by day 21. There was a significant increase in lymphocytes with Ag20cit at days 1 and 7 (**Fig. 2J**), which was not seen in BN rats (**Fig. 2E**). In SD rats, macrophages were also increased at day 7 for Ag20cit (**Fig. 2I**).

**Fig 3:**
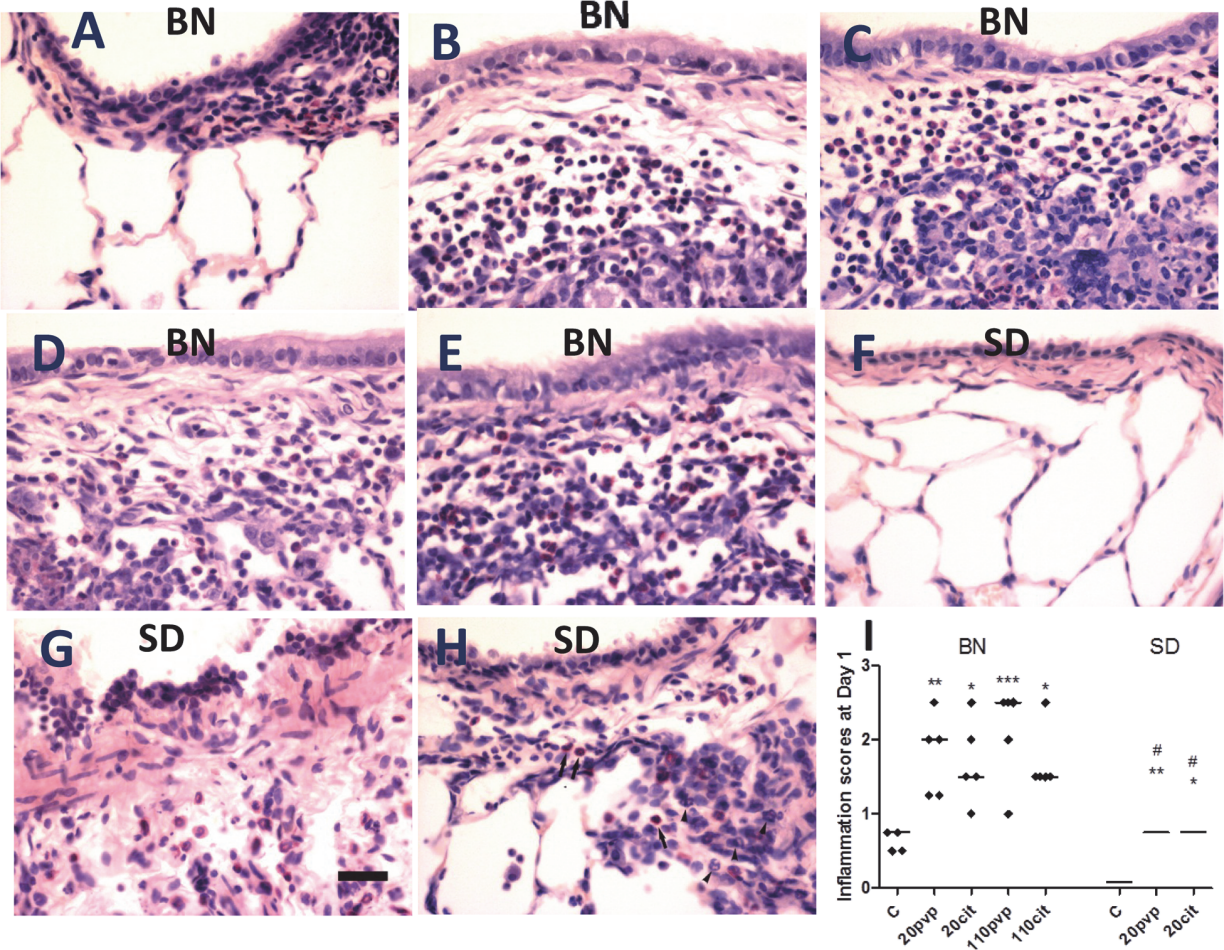
Representative haematoxylin- and carbo-chromotrope-stained lung sections from Brown-Norway (BN) rat lungs exposed to water only (Control) or to 20nm and 110 nm citrate- or pvp-capped silver nanoparticles (Panels A-E), and from Sprague-Dawley (SD) rats exposed to water only (Control) or to silver 20nm citrate- or pvp-capped nanoparticles (Panels F-H) at 24 hours after instillation. Compared to controls, inflammatory responses with neutrophils (arrow heads) are seen in both BN and SD rats after nanoparticle instillation. BN rats show the a predominance of eosinophils, while in SD rats, the inflammation is predominantly neutrophilic (Scale bar on Panel G is 10 μm, as used throughout). Panel I shows the distribution of inflammatory scores in these experiments with median bars shown for each experimental group. C is control rats instilled with water alone. *p<0.05; **p<0.01; ***p<0.001 compared to C; #p<0.05 compared to appropriate Brown-Norway group.

In the lungs, the inflammatory scores increased in both BN and SD rats at 24 hours but to a lesser extent in the SD than in the BN rat (**Fig. 3I**). The inflammatory response was predominantly neutrophilic, but the additional eosinophilic response was more prominent in the BN rat (**Fig. 3A to 3H**). These lung neutrophilic inflammation were similar to that observed in BAL fluid.

### Protein and monodialdehyde (MDA) levels in BAL

**Fig 4:**
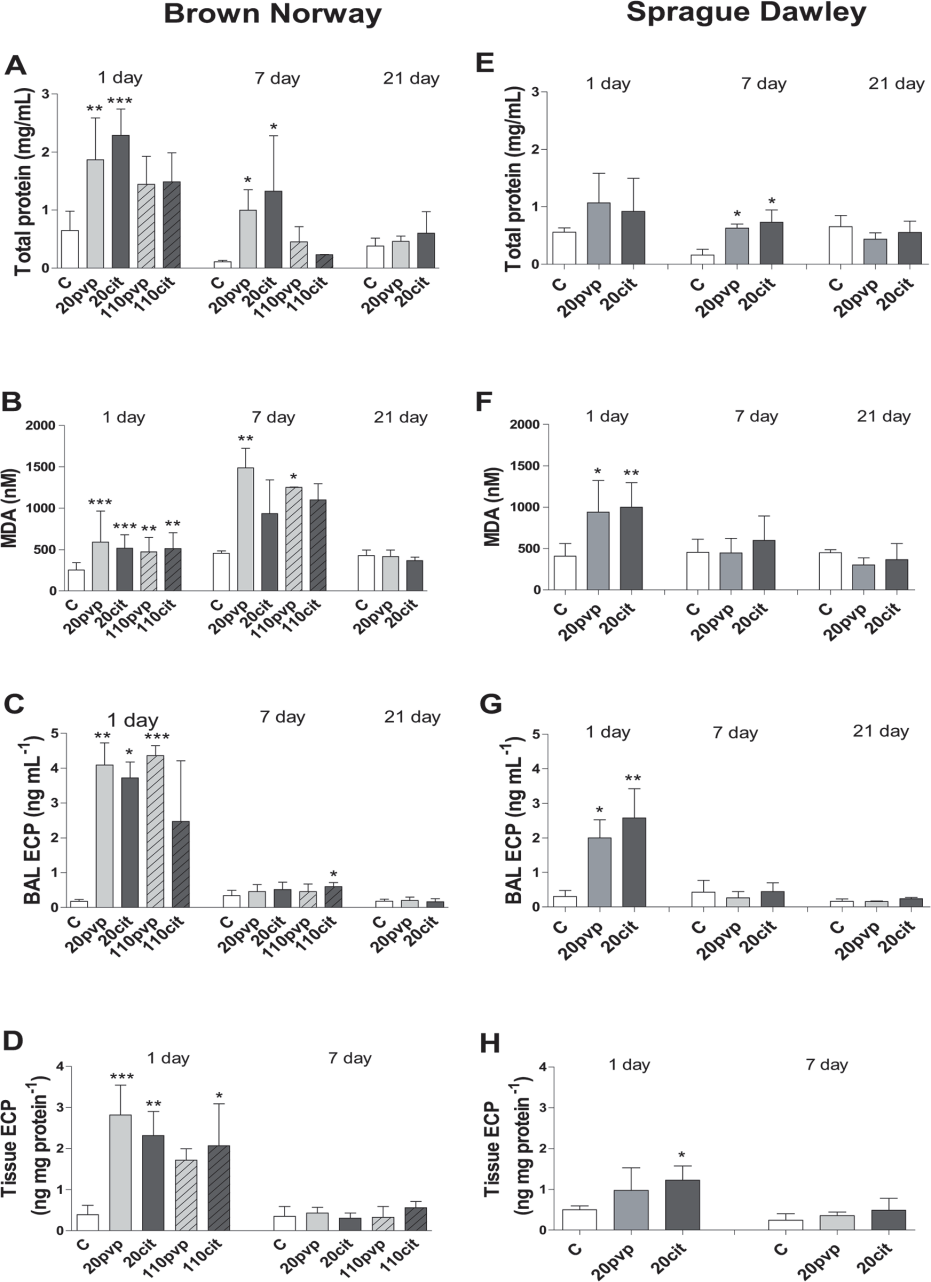
Effect of instillation of silver nanoparticles capped with citrate (cit) or polyvinlypyrrolidone (pvp) on BAL levels of protein, malonaldehyde (MDA) and eosinophil cationic protein (ECP), and on lung tissue ECP. Data is expressed as mean ± SD, n = 5–6 for each. *P<0.05, **P<0.001 and P<0.0001 compared to water control (C).

In BN rats, total BAL protein was increased by 3–4 fold at 24 hours, with a greater increase observed for Ag20citrate and Ag20pvp, that also persisted at day 7, but levels returned towards baseline at 21 days (**Fig. 4A**). In SD rats, total protein levels only transiently increased by 2-fold at day 7 for both 20 nm particles (**Fig. 4D**).

In the BN rat, there was a significant increase in total MDA, a by-product of lipid peroxidation, after instillation of all 4 AgNPs, at day 1 (**Fig. 4B**). At day 7, the level of MDA remained increased for the Ag20citrate and Ag20PVP NPs, but had returned to baseline by day 21. In SD rats, both 20 nm AgNPs increased the level of MDA at day 1 although levels had returned to baseline by day 7 (**Fig. 4F**).

### Eosinophillic Cationic Protein (ECP) in BAL and lung

In BN rats, BAL ECP levels increased following exposure to all AgNPs at day 1 except for Ag110cit, with levels returning to baseline by day 7, even though BAL eosinophils were persistently high at day 7 (**Fig. 4C**). ECP levels in BN rat lung tissue were elevated by all AgNP’s but had returned to baseline by day 7 (**Fig. 4D**). ECP was also detected in BAL from SD rats at day 1 after Ag20PVP and Ag20citrate NPs although this was lower than that measured in BN rats (**Fig. 4G**). There were no increases in ECP in the lung tissue of SD rats at 24 hours (**Fig. 4H**).

### KC, IL-5, IL-13, CCL11 and IgE levels in BAL

**Fig 5:**
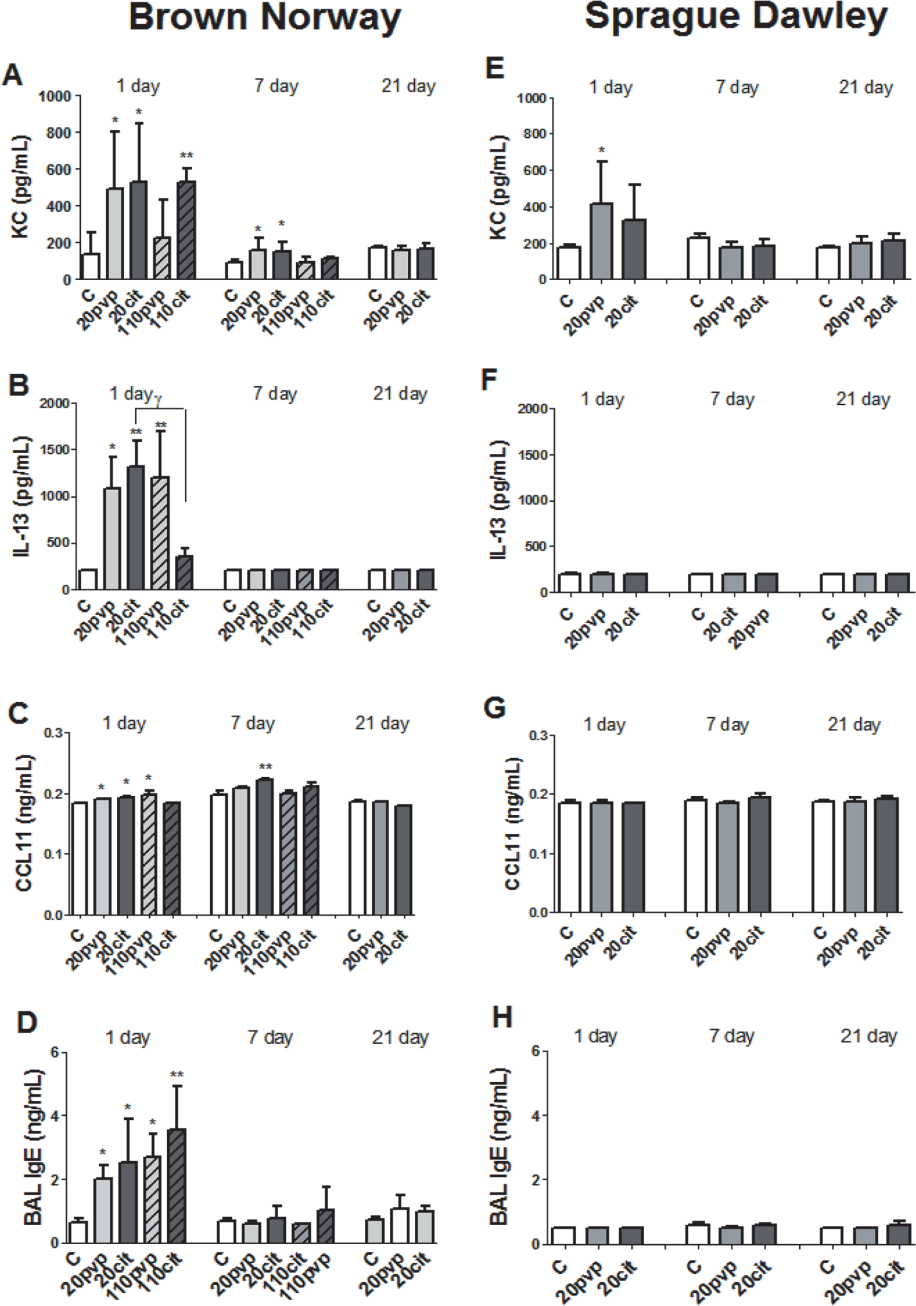
Levels of Keratinocyte Chemoattractant (KC), IL-13, CCL11 and IgE in broncho-alveolar lavage fluid at 1, 7 and 21 days after intratracheal instillation of nanosilver particles in Brown-Norway and Sprague-Dawley rats. Data is expressed as mean ± SD, n = 5–6 for each group. *P<0.05; **P<0.01compared to water control (C). ^*γ*^ p<0.05

In BN rats, KC levels were increased for the 20 nm AgNPs and for Ag110citrate at day 1 (**Fig. 5A**), with persistence of increased levels for the 20nm AgNP at day 7. Similarly, IL-13 levels were increased in BN rats only at 24 hours for both 20 nm AgNPs and Ag110pvp (**Fig. 5B**). CCL11 levels in BAL were increased for both 20 nm particles and the PVP-coated 110 nm particles at day 1 (**Fig. 5C**). Levels of IL-5 were not detectable (data not shown). BAL IgE was increased for all particles at 24 hours in BN rats (**Fig. 5D**). There was no increase in serum IgE, but baseline levels in BN rats were ~10–15 fold higher compared to SD rats (data not shown). For SD rats, there were no changes in these mediators in BAL apart from an increase in KC for Ag20PVP on day 1 (**Fig. 5E, F, G, H**).

### Gene expression of IFN^γ^, IL-5 and CCL11 in lung

Gene expression of IFN^γ^, IL-5 and CCL11 was performed in lung tissue for Ag20Citrate (Table 2). In BN rats, CCL11 expression increased 19.6-fold at day 1, disappearing by day 7. There were no changes in IL-5 or IFN-^γ^ expression in BN or SD rats.

**Table 2:** Gene expression in lung tissues of CCL11, IL-5 and IFNγ after instillation of 20 nm

		Brown-Norway rat	Brown-Norway rat	Sprague-Dawley rat	Sprague-Dawley rat
Cytokine	Day	Control	Ag20citrate	Control	Ag20Citrate
CCL11(eotaxin 1)	1	1.1 ± 0.2	20.0 ±5.4**	0.6± 0.3	1.3± 0.4
	7	1.2 ±0.5	2.8 ±1.7	0.6± 0.3	0.7± 0.1
IL-5	1	1.4 ±0.5	1.2± 0.5	1.0± 0.1	0.6± 0.1
	7	1.2 ±0.4	0.9 ±0.2	1.0 ±0.1	1.6 ±0.3
IFN^*γ*^	1	1.2 ±0.3	0.9 ±0.3	0.8 ±0.1	0.5 ±0.2
	7	0.9 ±0.3	0.9 ±0.2	1.1 ±0.2	1.5 ±0.2

silver nanoparticles at day 1 and day 7.

Data shown as fold-change and mean ± SD.

**P<0.01 compared to Control.

### Lung Resistance, dynamic compliance and bronchial hyperresponsiveness (BHR)

At day 1, Rrs increased by 2-fold in BN rats following exposure to all AgNPs, resolving by day 7 (**Fig. 5A**), with no increase in SD rats. In BN rats, Crs concomitantly decreased after exposure to all AgNPs, with persistence for Ag20citrate and Ag20PVP only at day 7 (**Fig. 5B**). At Day 21, there was only a persistent fall in Crs for Ag20Citrate In SD rats, there was a decrease in compliance at day 1 following Ag20Citrate exposure only (**Fig. 5F**).

**Fig 6:**
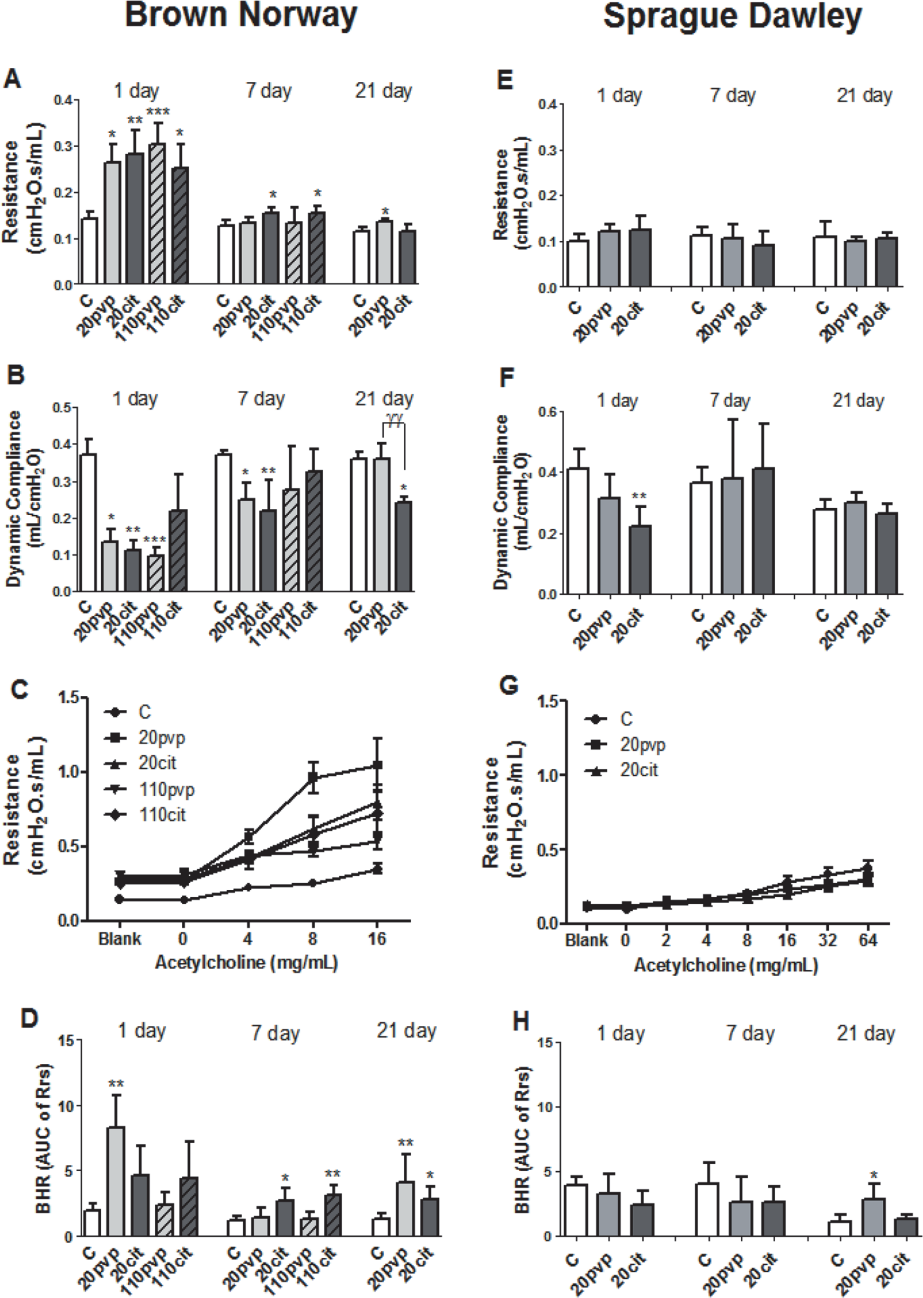
Effect of silver nanoparticles on lung function parameters at 1, 7 and 21 days after intratracheal instillation in Brown-Norway and Sprague-Dawley rats. Total lung resistance and dynamic compliance were measured at baseline and following nebulisation of increasing concentrations of acetylcholine (Panels A, B, E, & F). Mean concentration-response curves for each experimental condition are shown for Day 1 in Panel C for BN rats and in Panel G for SD rats. Bronchial responsiveness is represented by the area under the curve (AUC) of lung resistance versus concentration of acetylcholine (Panels D & H). Data is expressed as mean ± SD, n = 5–6 for each group. *P<0.05 **P<0.001, ***P<0.0001 vs the water control (C) within each time-point. ^*γγ*P^<0.01

In the BN rat, bronchial responsiveness was increased at day 1 after instillation of Ag20PVP and at day 7 after instillation of Ag20Citrate and Ag110Citrate (**Fig. 6D**). The mean concentration response curves at Day 1 are shown for each experimental group in **Fig. 6C** for BN rats and in **Fig. 6G** for SD rats. Interestingly, bronchial hyperrresponsiveness remained elevated at Day 21 for the Ag20pvp and Ag20citrate instillation in BN rats (**Fig. 6D**). In SD rats, there was a small increase in responsiveness for Ag20PVP at 21 days (**Fig. 6H**).

## Discussion

We investigated the impact of size and surface chemistry on the pulmonary toxicity of AgNPs by utilising well-dispersed AgNPs, with 20 and 110 nm diameters and two coating agents, PVP and citrate, designed to stabilise their dispersion. Dipalmitoylphosphatidylcholine (DPPC), the primary lipid constituent of lung lining fluid, improved the dispersion of these particles and retarded the dissolution of the 20 nm diameter-size AgNPs, which shared a greater tendency to dissolution. When instilled into rats, we found the greatest responses in the Brown-Norway rat compared to the Sprague-Dawley rat with a neutrophilic and more persistent eosinophilic response associated with bronchial hyperresponsiveness and an increased expression of IL-13 and CCL11 in the lungs. Regarding the influence of size and coating, we found mostly an effect of size on the neutrophilic inflammation with the smaller size having the biggest effect at the submaximal dose of nanoparticles we have chosen to use.

Nanoparticle toxicity has been linked to an increasing ratio of surface area to mass, shape, purity and associated changes in surface parameters such as reactivity, charge and solubility [29, 30]. Nanoparticle size is a major characteristic underlying deposition and fate in the lung. Smaller nanoparticles may cross cellular barriers more easily by modulating specific uptake and endocytic processes [31]. The increased bio-availability combined with a larger surface area may potentiate interaction with cellular organelles, increasing reactive oxygen species production, inflammation and cytotoxicity [32, 33]. To understand the effects of discrete nanoparticle sizes and coating agents on the pulmonary toxicity of silver, we studied the effect of non-agglomerated particles. We found that all the AgNPs tended to aggregate after incubation at physiological conditions, indicating that the nanoparticle dispersions were unstable under these conditions. With dipalmitoylphosphatidylcholine [DPPC], the primary lipid constituent of lung lining fluid, the particles remained better well-dispersed particularly for the 110nm AgNPs. DPPC absorbed onto the surface of the AgNPs to form a corona that could act as stearic stabiliser to prevent agglomeration [34]. Therefore, the 110 nm particles administered intratracheally would be expected to behave as discrete nanosize particles through stabilisation by DPPC in the lung liquid layer. The smaller 20nm AgNPs would form less stable agglomerates, potentially increasing the surface area available for biological interaction with proteins, lipids and inorganic ions in the lung fluid milieu.

A potential mechanism for the increased toxicity of smaller AgNPs is dissolution and release of silver ions following oxidation of the particle surface [35, 36]. To model the interactions with the lung lining fluid *in vivo*, we characterised the dissolution of the 20 and 110 nm nanoparticles in both water and DPPC. We found higher rates of dissolution for the 20 nm particles in water with a reduced dissolution rate in DPPC suggesting that surfactant interaction stabilised the surface conditions, preventing oxidation, which would otherwise lead to the release of Ag^+^ ions. The rate of dissolution for the 110 nm particle was negligible, suggesting that the release of Ag^+^ ions may play an important role in the increased toxicity of the 20 nm nanoparticles.

In the BN rat, the smaller 20 nm AgNP induced greater neutrophilic inflammatory effect compared with the 110 nm AgNPs, together with a greater increase in total BAL protein, indicating that the blood/alveolar epithelial permeability barriers have been disrupted with a resulting increase in leaked protein content from the microvasculature. These differences may be related to their lower stability, greater solubility and the increased bioavailability of the 20nm compared with the 110nm AgNPs are therefore potential mechanisms for the increased toxicity of the smaller particles seen in the Brown-Norway rats. We showed that the instillation of silver ions at a dose calculated from our characterisations of the 20 nm AgNPs in DPPC, did not induce any inflammatory or lung response in SD rats indicating that the dissolving Ag^+^ ions in the lung lining fluid, were unlikely to be contributing to the *in vivo* toxicity of these AgNPs. However the increased dissolution rate of the 20 nm AgNP may contribute to their greater toxicity following uptake in lung cells and tissues. On the other hand, there was no difference in the eosinophilic response both in terms of eosinophil numbers and the levels of its product, eosinophil cationic protein, and levels of oxidative stress as indicated by malondialdehyde levels, between the 20 nm and the 110 nm sized particles, which may indicate that the mechanisms underlying these effects may be rate-limited.

The citrate-coated 20 and 110nm AgNPs induced a greater and longer duration of neutrophilic response and BHR, compared with the PVP-coated nanoparticles suggesting that the loss of stability of the capping agents and associated dissolution could be influencing these responses. The magnitude of the inflammatory responses measured in this study with either PVP- and citrate-coated nanoparticles are far in excess of those reported for *in vivo* studies with inhaled uncoated nanoparticles [17–19]. This suggests that stabilisation of the AgNPs by coatings prior to interaction with surfactant molecules in the lung lining fluid may underlie this greater inflammatory response we observed.

There were significant differences observed betweenthe 2 rat strains in their response to instillation of the 20nm AgNPs. Brown-Norway rats exhibited an early bronchoconstrictor response with BHR and an early neutrophil and eosinophil recruitment. In contrast, in the Sprague-Dawley rats, there was a predominant early neutrophilic response at 24 hours, with a minor degree of eosinophilia. Neutrophillia was equally prominent in both rat strains indicating that it is a well-known response to the surface area-driven effects of AgNPs [29]. The eosinophilic responses in Brown-Norway rats were accompanied by an increase in IL-13 and CCL11 levels in the lungs but not of IL-5 indicating that the eosinophilic response could be driven by these cytokines [37]. The early influx of eosinophils in the Brown Norway rat also correlated with increase in eosinophil cationic protein, a marker of eosinophil degranulation, in bronchoalveolar lavage fluid and tissues [38, 39]. We did not study the 110 nm silver particles in the Sprague-Dawley rat because we were interested in determining the differences in pulmonary response between the 2 rat species and the 20 nm AgNPs were more toxic than the 100 nm AgNPs in the Brown-Norway rat. We therefore chose to study only the 20 nm AgNPs in the Sprague-Dawley rat.The development of an eosinophilic lung reponse to instillation of AgNPs in BN rats is a new finding, but other nanoparticles such as zinc and copper oxides have been reported to induce a neutrophilic and eosinophilic response in Wistar rats [40, 41]. The inflammatory effect of the zinc and copper oxide was reported to be due to the nanoparticles rather than to the solubilised ions. The Brown-Norway rat has been used as a model of allergen-induced BHR, mimicking features of human allergic asthma, including an early and late bronchoconstrictor response, an increase in antigen-specific IgE following active immunization, eosinophilic airway inflammation, and bronchial hyperresponsiveness following allergen challenge [42, 43]. In contrast, Sprague-Dawley rats seldom develop an early airway response or detectable serum-specific IgE with active immunisation, and also bronchial hyperresponsiveness [26, 27, 44]. One potential explanation of these contrasting differences is that alveolar macrophages isolated from Brown Norway rats produce more nitric oxide and Th2-type cytokines (e.g. IL-10 and IL-13) and less Th1-type cytokines (e.g. TNF) than those from the Sprague-Dawley rat [44]. Similar differences in response to an intratracheal instillation of AgNPs between the 2 rat strains are observed, particularly in the development of an eosinophilic response and of bronchoconstriction and bronchial hyperresponsiveness. In addition, the transient increase in bronchoalveolar lavage IgE levels observed only in the Brown-Norway rat after AgNP instillation would also support the induction of Th2 cytokines such as IL-4 and IL-13, which are involved in inducing IgE isotype switch in B-cells. Our data support the notion that AgNPs are capable of inducing the asthmatic diathesis in predisposed individuals such as those with asthma or with an existing Th-2 bias. The relevance of these findings is that AgNPs may potentially induce an eosinophilic response in the airways of subjects with an asthmatic predisposition or with an atopic background, and also increase the chance of an asthmatic response if ever they became exposed to these particles.

In summary, we have shown that the 20nm versus the 110 nm size were more proinflammatory in terms of neutrophil influx but not in terms of eosinophilic influx, but there was little difference between the citrate-capped versus the PVP-capped AgNPs. AgNPs also caused a disruption in the blood/alveolar epithelial permeability barrier, oxidative stress and activation of eosinophils, with release of cytokines KC and IL-13 and IgE. They also induced an increase in lung resistance and an increase in bronchial responsiveness only for the 20 nm AgNPs. Importantly, AgNPs can induce pulmonary eosinophilic and neutrophilic inflammation with bronchial hyperresponsiveness, features characteristic of asthma, particularly in BN rats. Our data indicate that exposure to AgNPs may lead to induction of the asthma diathesis.
